# High Voltage Electrochemiluminescence (ECL) as a New Method for Detection of PAH During Screening for PAH-Degrading Microbial Consortia

**DOI:** 10.1007/s11270-015-2532-1

**Published:** 2015-07-24

**Authors:** Justyna Staninska, Zuzanna Szczepaniak, Krzysztof Staninski, Jakub Czarny, Agnieszka Piotrowska-Cyplik, Jacek Nowak, Roman Marecik, Łukasz Chrzanowski, Paweł Cyplik

**Affiliations:** Department of Biotechnology and Food Microbiology, Poznań University of Life Sciences, Wojska Polskiego 48, 60-627 Poznań, Poland; Institute of Food Technology of Plant Origin, Poznań University of Life Sciences, Wojska Polskiego 31, 60-624 Poznań, Poland; Department of Rare Earth, Faculty of Chemistry, Adam Mickiewicz University, ul. Umultowska 89b, 61-614 Poznań, Poland; Institute of Forensic Genetics, Al. Mickiewicza 3/4, 85-071 Bydgoszcz, Poland; Institute of Chemical Technology and Engineering, Poznań University of Technology, Pl. M. Skłodowskiej-Curie 2, 60-965 Poznań, Poland

**Keywords:** Biodegradation, High voltage electrochemiluminescence (ECL), Metagenomics analysis, PAH

## Abstract

The search for new bacterial consortia capable of removing PAH from the environment is associated with the need to employ novel, simple, and economically efficient detection methods. A fluorimetric method (FL) as well as high voltage electrochemiluminescence (ECL) on a modified surface of an aluminum electrode were used in order to determine the changes in the concentrations of PAH in the studied aqueous solutions. The ECL signal (the spectrum and emission intensity for a given wavelength) was determined with the use of an apparatus operating in single photon counting mode. The dependency of ECL and FL intensity on the concentration of naphthalene, phenanthrene, and pyrene was linear in the studied concentration range. The biodegradation kinetics of the particular PAH compounds was determined on the basis of the obtained spectroscopic determinations. It has been established that the half-life of naphthalene, phenanthrene, and pyrene at initial concentrations of 50 mg/l (beyond the solubility limit) reached 41, 75, and 130 h, accordingly. Additionally, the possibility of using ECL for rapid determination of the soluble fraction of PAH directly in the aqueous medium has been confirmed. Metagenomic analysis of the gene encoding 16S rRNA was conducted on the basis of V4 hypervariable region of the 16S rRNA gene and allowed to identify 198 species of bacteria that create the S4consortium. The consortium was dominated by *Gammaproteobacteria* (78.82 %), *Flavobacteria* (9.25 %), *Betaproteobacteria* (7.68 %), *Sphingobacteria* (3.76 %), *Alphaproteobacteria* (0.42 %), *Clostridia* (0.04 %), and *Bacilli* (0.03 %).

## Introduction

Polycyclic aromatic hydrocarbons (PAHs) are unique environmental contaminants, which are constantly released through both naturally occurring processes (forest fires) and anthropogenic activity (burning of fuels in mechanic vehicles, house heating systems, thermal methods of waste utilization). The highest concentration of PAH contamination is usually detected in town centers, along the highways, in the vicinity of production plants and coal-fired power plants. These compounds exhibit toxic, mutagenic, and cancerogenic properties (Johnsen and Karlson 2007). PAHs are composed of at least two aromatic rings, which makes them resistant to nucleophilic attacks. Moreover, their low water solubility and high partition coefficient between the solid and liquid phase contribute to their accumulation in the solid parts of soil. All the above-mentioned properties concerning their chemical composition, as well as other factors influencing their bioavailability for microorganisms, make them hardly biodegradable substances in the terrestrial environment (Johnsen et al. 2005). The age of PAH contaminations accumulated in urban agglomeration soils is estimated to be over 100 years. Even though the microorganisms capable of biodegrading PAH are present in soil, the concentration of PAH remains at a constant level throughout decades. This is most likely caused by the limited bioavailability of PAH to microorganisms. The bioavailability can be described by two factors: chemical activity and assimilation. These factors influence the progress of spontaneous processes, such as diffusion and partitioning of PAH between the solid and liquid phase, thus controlling PAH transport through cell membranes. This is caused by the gradient of chemical activity between the inner parts of cell and the outside environment (Johnsen et al. 2005). Due to the above-mentioned phenomena, the studies should not only be based on the chemical characteristics of target contaminants, but also on the investigation of mechanisms describing their uptake by microorganisms from the environment, which is more significant in terms of biodegradation processes.

The qualitative and quantitative analyses are a crucial issue associated with the screening of microorganisms capable of efficient PAH biodegradation. The currently employed analytical methods allow for the separation and precise determination of PAH compounds. These methods include gas or liquid chromatography or solid-phase extraction techniques (Chen et al. 2007; Pillai et al. 2005) with subsequent fluorescent or electrochemical detection (Peltonen and Kuljukka 1995; Chen et al. 1996; Jiji et al. 1999; Kershaw and Fetzer 1995). Unfortunately, these methods rely on the use of toxic solvents and require filling the columns with portions of activated sorbents for each analysis. Hence, there is a need to develop a new, simple test with high diagnostic value, which would be both economically efficient as well as consistent with the green chemistry idea (by reducing the use of hazardous organic solvents). The use of high voltage electrochemiluminescence (ECL), which occurs on the metal/semiconductor/solvent interfacial boundary, seems to be a promising solution.

The electrochemical experiments carried out with the use of an aluminum electrode coated with a nanolayer of aluminum oxide indicate that this method can be successfully employed for determination of biologically active organic compounds (Kulmala et al. 2002; Eskola et al. 1995; Ala-Kleme et al. 2006) as well as inorganic compounds (Staninski 2010; Staninski and Lis 2008). The aim of this study was to evaluate the usefulness of combined ECL and FL methods for determination of biodegradation kinetics of selected PAH compounds in aqueous solutions.

## Materials and Methods

### Isolation of Microorganisms

The S4 bacterial consortium isolated from the area of Polish Carpathian mountains was used for the evaluation of the biodegradation potential. The isolation of microorganisms was carried out according to the procedure described in Owsianiak et al. (2009).

### Culture Conditions

Loosely capped 250-ml Duran-Schott bottles contained 50 ml of mineral medium. The initial inoculum was adjusted to an optical density of 0.10 ± 0.03 at 600 nm, and the bacteria were cultured at 22 °C and 100 rpm. The composition of the mineral medium used during the experiments was as follows (g/l): KH_2_PO_4_ 2.8, NaCl 0.5, NH_4_Cl 1.0, MgSO_4_ × 7H_2_O 0.01, FeSO_4_ × 7H_2_O 0.001, MnSO_4_ × 4H_2_O 0.0005, ZnCl_2_ 0.00064, CaCl_2_ × 6H_2_O 0.0001, BaCl_2_ 0.00006, CoSO_4_ × 7H_2_O 0.000036, CuSO_4_ × 5H_2_O 0.000036, H_3_BO_3_ 0.00065, H_2_MoO_4_ 0.005, EDTA 0.001, and HCl 37 % 0.0146 ml/l. The initial concentration of naphthalene, phenanthrene, and pyrene (50 mg/l) was well beyond their respective water solubility limits, which allowed to achieve a microemulsion system. Due to the high ionic strength of the mineral medium, the system was susceptible to coagulation processes; hence, agglomerates with higher molecular mass were formed, and a suspension of macromolecules was obtained.

### Analytical Methods

#### Genetic Identification of Microorganisms

A 5-ml aliquot of cultured bacteria was centrifuged for 15 min at 10,000 × *g*, and the pellet was washed twice with sterile water. DNA was extracted from the pellet using EZ1 DNA Tissue Kit (Qiagen) and EZ1 DNA Bacteria Card (Qiagen) on EZ1 workstation. Metapopulation analysis of the V4 region of 16SrRNA was performed as previously described by Caporaso et al. (2011, 2012). Data was analyzed using Analysis software version 2.4.60.8 (Illumina).

#### Fluorescence Analysis

The fluorimetric and electrochemiluminescent methods were employed to evaluate the biodegradation efficiency of naphthalene, phenanthrene, and pyrene. The fluorescence determination method is based on highly efficient quantum fluorescence of hydrocarbons possessing conjugated aromatic rings (Schwarz and Wasik 1975). In order to determine the excitation and emission wavelengths, the excitation and fluorescence spectra for naphthalene, phenanthrene, and pyrene were obtained by using commercial PAH standards in hexane at a concentration of 10^−4^ mol/l. The wavelength of excitation radiation (λ_exc_) for naphthalene, phenanthrene, and pyrene was at 275, 348, and 340 nm, accordingly. Afterward, the PAH standard solutions in hexane were used to carry out measurements of fluorescence intensity at analytical wavelengths of λ_em_ = 323, 384, and 386 nm, and the calibration curves were obtained.

Since hydrocarbons in the solid state also exhibit intense fluorescence (the undissolved molecules in the exciting beam of the fluorimeter may considerably interfere with the measurement), a threefold extraction of samples with the use of 100 ml of hexane was carried out (5 min). The measurements were carried out using a Hitachi F-7000 spectrofluorometer.

### Electrochemiluminescent Analysis (ECL)

**Fig. 1:**
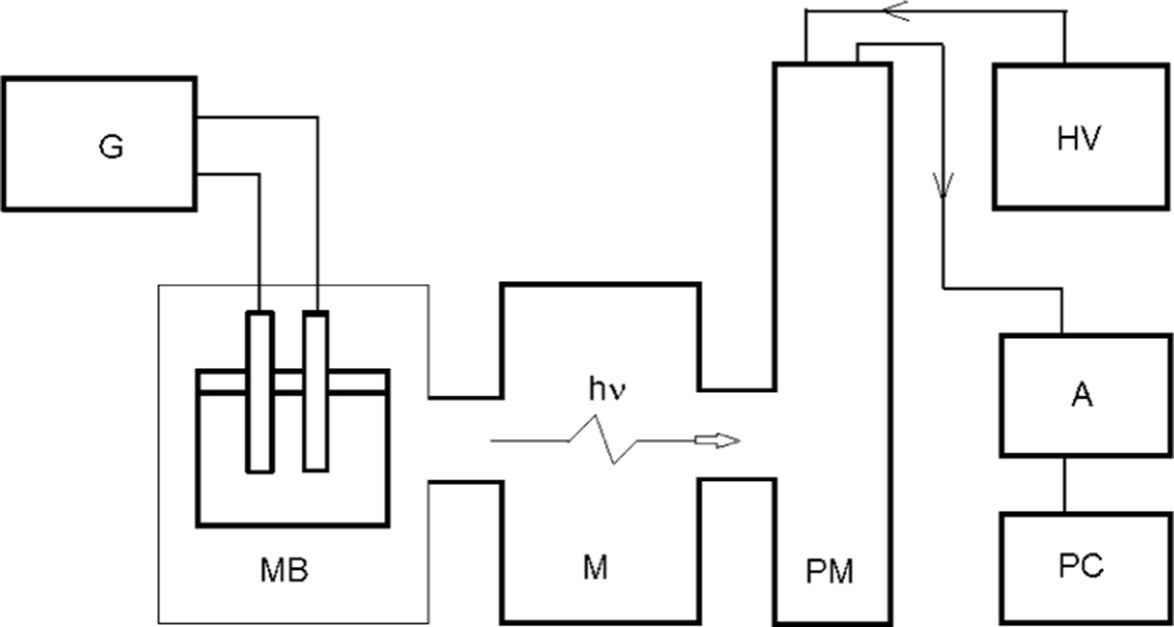
Measurement setup for high voltage electrochemiluminescence investigation in two-electrode sysytem; *G* coulostatic pulse generator, *MB* measurement cell, *M* monochromator TRIAX-180, *PM* photomultiplier Hamamatsu R-636, *HV* high voltage power supply, *A* amplifier HAMAMATSU C5594-34, *PC* counting module Advantech PCI-1780U with personal computer

The ECL analysis was conducted using the authorized measurement setup. Figure 1 presents a scheme of the measuring setup with its most important elements. For the sake of clarity, such elements, as low voltage suppliers (5 V, 12 V, and 15 V) to work with amplifiers and cooling system, have been omitted. The measuring chamber (MB) is the first element of the system. It is a lightproof box with a regulated size outflow slit and a parabolic mirror.The sample is mounted at the focus of the mirror. The chamber has a sample holder, ports to connect the flow-though cell working, and a connector to an external source of potentiostatic and coulostatic generator (G) of rectangular pulses for excitation of electrochemiluminescence. The luminescence generated in the process of ECL is directed by the optical track to a TRIAX-180 monochromator (M). The monochromator controlled via the computer application permits recording of the ECL spectra with a maximum resolving power of 0.3 nm in the range 180–800 nm. Next, the signal is directed to photomultiplier Hamamatsu R-636 (PM), which works using single photon counting method, and cooled to −15 °C. The electric pulses from the photomultiplier are amplified at a C5594-34 amplifier (A) and recorded by a PC via a card Advantech PCI-1780U.

In order to determine the analytical wavelengths, the electrochemiluminescent spectra of aqueous solutions of naphthalene, phenanthrene, and pyrene were obtained using the Al/Al_2_O_3_ electrode. The electrochemiluminescence process was generated in a dual electrode system, with an aluminium electrode coated with a nanolayer of aluminium oxide as the working electrode and a platinum counter electrode. The postcultivation samples were centrifuged (5000 rpm, 2 min), and afterward, 5 ml of the supernatant was injected into the measuring dish (MD) in the measuring chamber (MB). Excitation of aromatic compounds (PAH) occurred via electric impulses with an amplitude of 40 V and a frequency of 30 Hz with a single impulse charge of 70 μC.

### Statistical Methods

The errors in the intensity of electrochemiluminescence and fluorescence during the determination of the calibration curves were assumed as the standard deviation of the arithmetic mean from 12 measurements. Measurement of uncertainty during the determination of hydrocarbon concentrations was estimated using the exact differential method.

## Results and Discussion

### Evaluation of Naphthalene, Phenanthrene, and Pyrene Biodegradation Efficiency with the Use of the Fluorescence Method

**Fig. 2:**
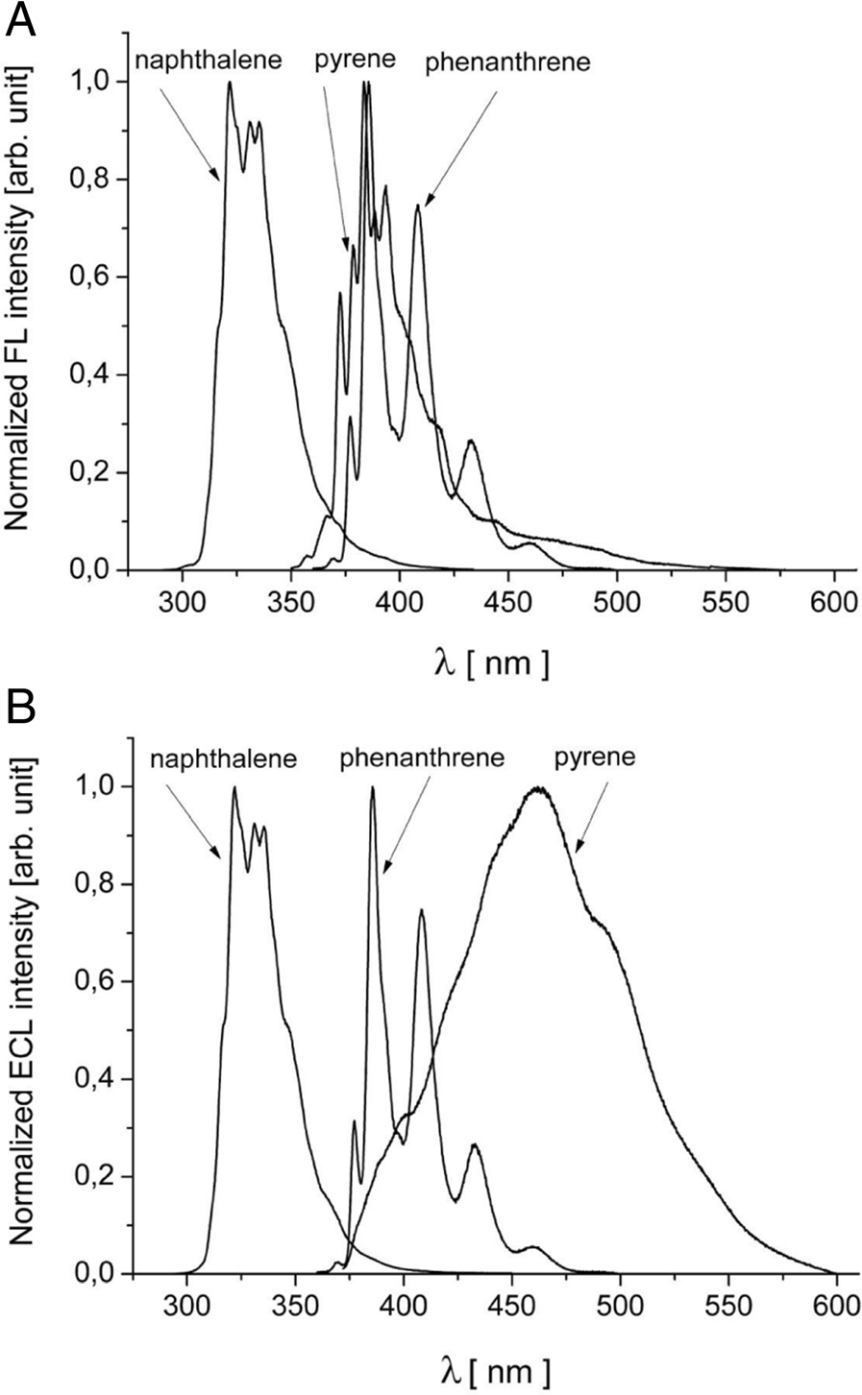
Normalized fluorescence spectra (**a**) of naphthalene, phenanthrene, and pyrene solutions in hexane. The employed excitation wavelengths λ_exc_ were at 275, 348, and 340 nm, accordingly. Normalized electrochemiluminescence spectra of naphthalene, phenanthrene, and pyrene on an Al/Al_2_O_3_ electrode in aqueous solutions at concentrations of 0.5, 0.3, 0.1 mg/dm^3^, accordingly. The excitation process was carried out with the use of rectangular impulses with an amplitude of 40 V and a frequency of 30 Hz with a single impulse charge of 70 μC

In order to improve the sensitivity of determination for naphthalene, phenanthrene, and pyrene, an extraction was carried out using hexane. This allowed for elimination of water particles, which is crucial, since water may efficiently deactivate the excited state of aromatic hydrocarbons. This is also true for oxygen; therefore, oxygen was removed from all samples by flushing with an inert gas (argon). On the basis of excitation spectra of the studied PAH compounds, the excitement wavelength for naphthalene, phenanthrene, and pyrene was determined. The obtained fluorescence spectra are shown in Fig. 2a.

**Fig. 3:**
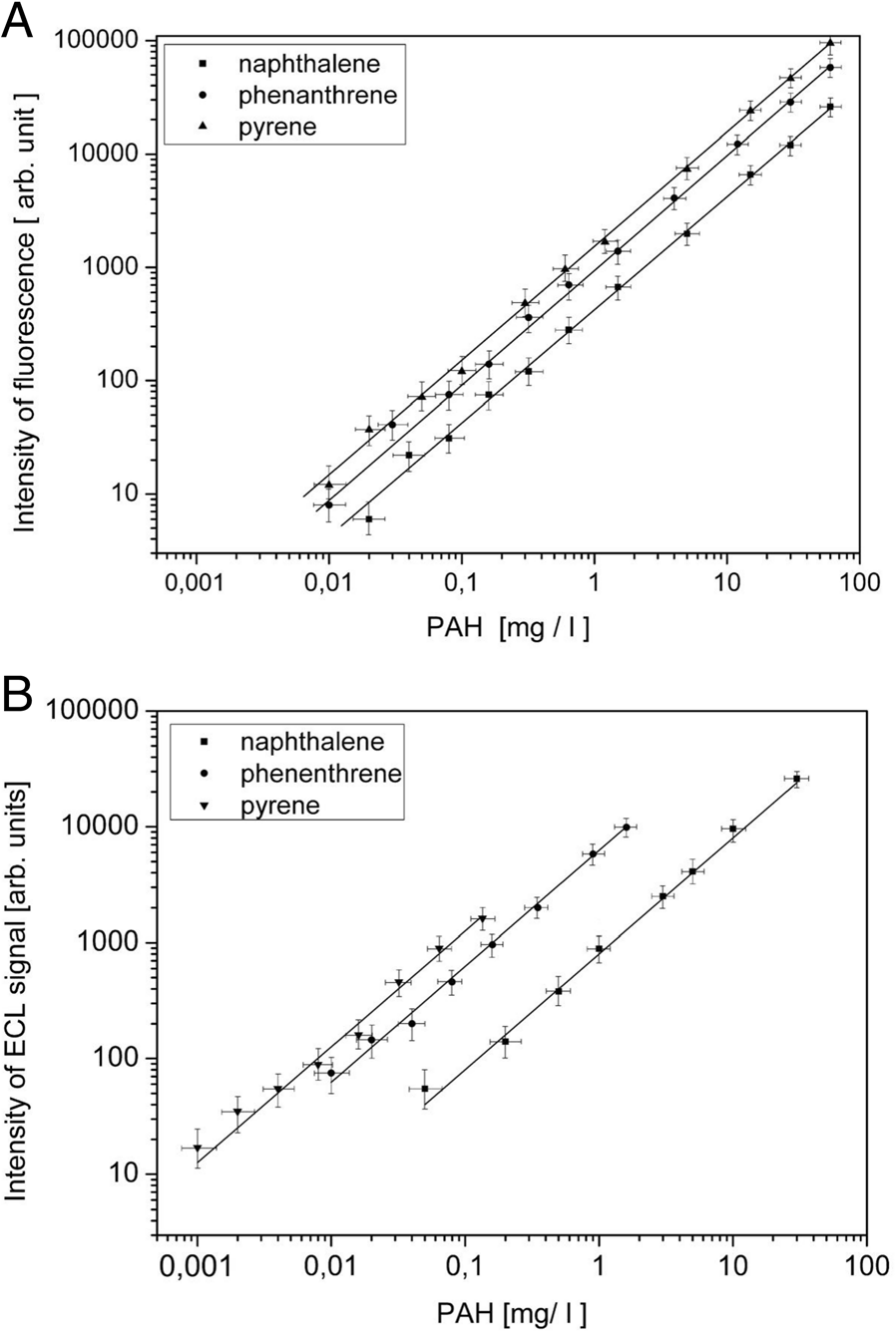
Calibration curves for fluorimetric determination of the studied PAH compounds in hexane (**a**), electrochemiluminescent determination of the studied PAH compounds in water (**b**)

The intensity of absorption and fluorescence was normalized to value 1 relative to the value of maximum intensity in order to improve the comparison of bands specific for the studied compounds. The determinations were carried out in the range of ultraviolet wavelengths for bands characterized by the highest emission intensity. The structural similarities of PAH contribute to adequate similarities in the range of electron transition energy; hence, the qualitative determination of each compound in the mixture was challenging. On the basis of the obtained fluorescence spectra, the emission wavelengths (λ_em_) of 275, 340, and 348 nm were selected for determination of naphthalene, phenanthrene, and pyrene, accordingly. By using standard compounds in the concentration range of 0.01–70 mg/dm^3^, a linear dependence of fluorescence intensity was obtained for phenanthrene and pyrene. For naphthalene, the linear dependence was obtained in the concentration range of 0.02–70 mg/dm^3^. The standard curves are presented in Fig. 3a. The linear correlation coefficients (*R*) for each curve were >0.99.

**Fig. 4:**
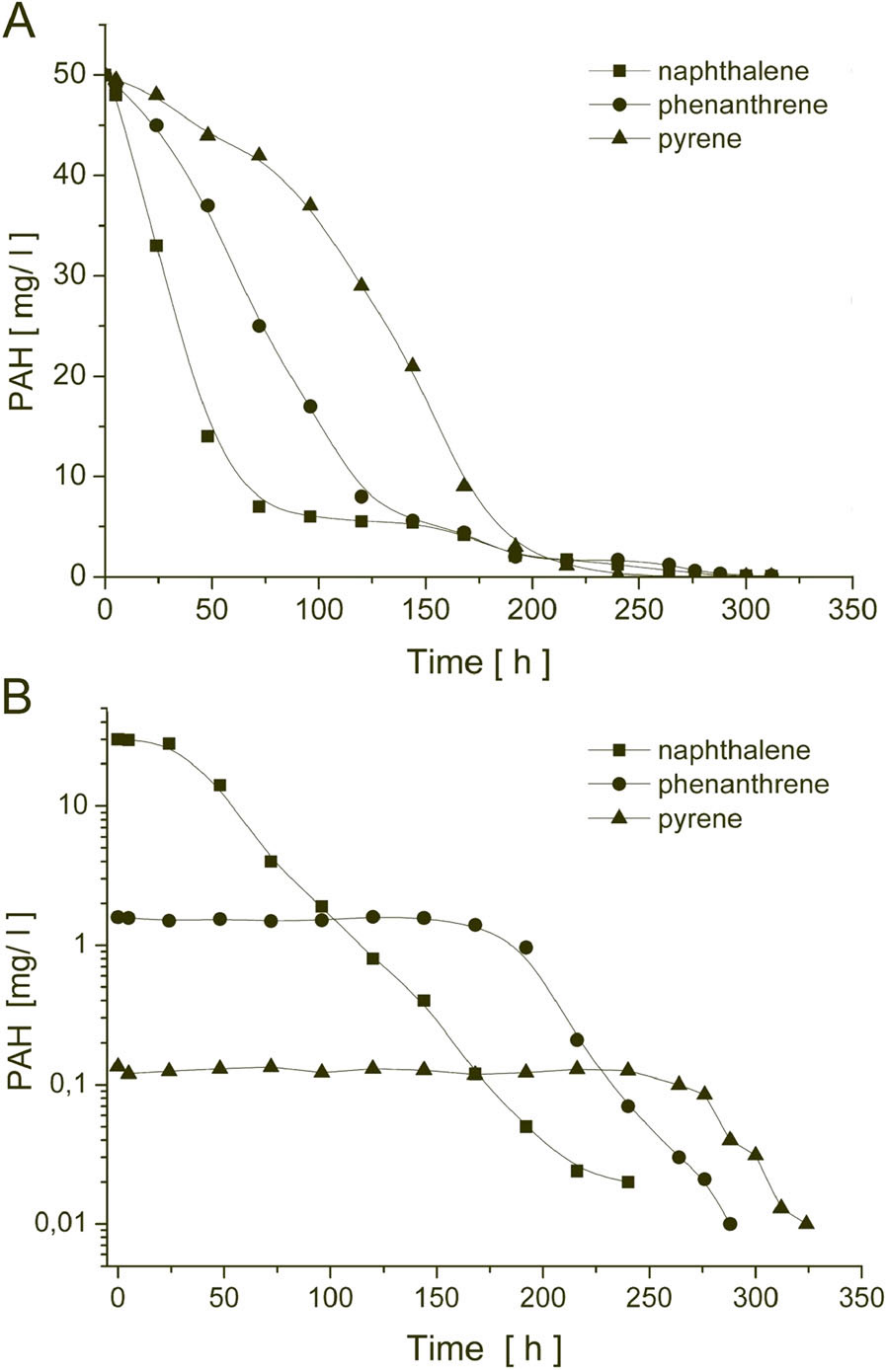
Changes in the PAH concentration during biodegradation in the aqueous environment on the basis of fluorimetric (**a**) and electrochemiluminescent (**b**) determination

Bioavailability of PAH is considered as one of the major factors, which determine their biodegradation rate in the environment (Haritash and Kaushick 2009). In aqueous systems, the crucial issue was the dispersion of the substrate, since its bioavailability increased with improved solubility and fragmentation. In the studied samples, the alcohol solutions of PAH dosed into the aqueous phase formed emulsions; however, in the presence of an electrolyte, the compounds started to coagulate and form macrometric particles, which decreased the bioavailability. In this case, the highest bioavailability was exhibited by hydrocarbons with the highest water solubility and the lowest number of aromatic rings in the chemical structure. The biodegradation kinetics of the analyzed PAH were estimated on the basis of the reduction of their concentration in time and presented in Fig. 4a. The microbial degradation of a given PAH compound occurred at a different rate. Among the studied compounds, naphthalene was most susceptible to biodegradation, most likely due to its highest solubility (~30 mg/dm^3^). The biodegradation rate of phenanthrene was lower, similar to its solubility (~1.6 mg/dm^3^), whereas the biodegradability of pyrene was the lowest, which can be attributed to its low water solubility (0.135 mg/dm^3^). After 230–250 h, most of the PAH compounds were decomposed. Similar observations, which confirm the limiting influence of solubility on the biodegradation of PAH, were reported by Tikilili and Nkhalambayausi-Chirwa (2011). The authors established that low molecular weight PAHs were characterized by a higher biodegradation rate compared to high molecular weight PAH. However, it should be noticed that physicochemical properties are not the only determinant when considering the biodegradation kinetics. Therefore, the biodegradation rate cannot be unequivocally determined solely on the basis of the number of aromatic rings in the structure or solubility. It seems that the composition of the bacterial population and its catabolic potential may also be a significant factor, at least in simple aqueous systems. The results obtained by Knightes and Peters (2003) revealed that the biodegradation rates of different PAH compounds with various molecular weights (including naphthalene, phenanthrene, and pyrene) by an environmental consortium (with different composition of species compared to the one employed in this study) were similar. Moreover, the earlier study of Boldren et al. (1993) with the use of *Mycobacterium* sp. confirmed that although the biodegradation rate of phenanthrene, fluoranthene, and pyrene is associated with their physicochemical properties, the microbial growth is independent. On the basis of this statement, it can be concluded that biological systems are highly complex and species-specific.

### Evaluation of Naphthalene, Phenanthrene, and Pyrene Biodegradation Efficiency with the Use of the ECL Method

In order to determine the changes in the concentrations of the studied PAH, a novel method based on the high voltage electrochemiluminescence on an aluminum electrode was employed (Kulmala et al. 2002; Eskola et al. 1995; Ala-Kleme et al. 2006). In this method, the molecules with luminescent properties are excited in the aqueous medium based on secondary reactions of radical oxidation and reduction. Only the dissolved compounds become excited; hence, under specific conditions, the ECL intensity is proportional to the concentration of the compound subjected to excitation. The ECL spectra of aqueous solutions of naphthalene, phenanthrene, and pyrene are presented in Fig. 2b. For an easier comparison, the intensity of particular bands was normalized to value 1 relative to the value of the band with maximum intensity, similar to the results obtained for fluorescence spectra. The electrochemical conditions of the ECL process were selected on the basis of experimentation in order to achieve maximum efficiency of emission. The determined bands with maximum ECL intensity for naphthalene, phenanthrene, and pyrene were found at 323, 386, and 465 nm, accordingly. Comparison of fluorescence spectra (Fig. 2a) and electrochemiluminescence spectra (Fig. 2b) revealed that the maxima values of FL and ECL differed for the studied hydrocarbons. This fact may be attributed to the difference in the polarity of the employed solvents—hexane and water. The highest discrepancies were observed for pyrene, since its Π → Π^*^ electron transitions are dependent on the dielectric constant of the surrounding environment.

A strong bathochromic shift as well as the reduction of the oscillation structure can be observed in the spectrum. The present hydrocarbons are excited in ECL conditions mainly by oxidation-initiated redox pathways. At first, the organic molecule is oxidized by hydroxyl radicals generated by reduction of dissolved oxygen (Kulmala et al. 2002; Eskola et al. 1995; Ala-Kleme et al. 2006; Staninski 2010; Staninski and Lis 2008). The next step is the reduction of the oxidized molecule by “hot” or hydrated electrons, resulting in the excitation of hydrocarbons. After this, the process of deactivation is analogous to the photoluminescence of these PAH molecules.

In order to determine the influence of the solution components on the processes of deactivation of excited PAH, the mineral medium solutions used during the experiments were studied as potential free radical scavengers. It was established that the salts used in the mineral medium did not influence the efficiency of PAH electrochemiluminescence processes in a notable manner. The highest contribution to deactivation of emission processes was observed for Fe^3+^ and Cu^2+^ ions; however, their trace amounts as microelements have no practical influence on the obtained ECL results. Similar to the FL method, the calibration curves describing the dependency of ECL intensity and the concentration of PAH were obtained (Fig. 3b). The detection limit of the studied PAH compounds in aqueous solutions was limited by their solubility. Linear dependencies of ECL intensity and the concentration at the selected wavelengths for naphthalene, phenanthrene, and pyrene were obtained in the range of 0.05–30, 0.01–1.6, and 0.001–0.12 mg/dm^3^, accordingly. The changes in ECL intensity were strongly correlated with the decrease in PAH concentrations determined by the FL method. When the concentration of the given hydrocarbons falls below the solubility limit (naphthalene 30 mg/dm^3^, phenanthrene 1.6 mg/dm^3^, and pyrene 0.135 mg/dm^3^), a change in the ECL signal occurs. The unchanged ECL signal for phenanthrene and pyrene (150 and 275 h, accordingly) may be attributed to the fact that their concentrations remained constant during the biodegradation process, due to exceeding their respective solubility limits in water (Fig. 4b). Their removal by means of microbial biodegradation was compensated by the dynamic dissolution of the solid hydrocarbon fraction present in the system. Comparison of biodegradation curves (Fig. 4) for naphthalene and phenanthrene (after 190 h) revealed that their slopes are similar. This corresponds to the fact that decomposition of naphthalene and phenanthrene in the aqueous medium occurred at a similar rate.

Electrically generated chemiluminescence as a method for determination of water soluble PAH may potentially be useful during screening of microorganisms capable of effective PAH degradation. This method could be employed as the first step of improving the bioremediation technologies. Another application possibility is associated with the monitoring of such compounds during wastewater treatment processes. It should be highlighted that according to the European Union legislation, the maximal concentration of PAH compounds in drinking water should be strictly controlled due to their cancerogenic properties (Council Directive 98/83/EC on the quality of water intended for human consumption). As a result, there are numerous studies dedicated to the evaluation of efficiency and optimization of treatment processes (Nowacka and Włodarczyk-Makuła 2013; Muff and Søgaard 2010). A rapid and simple detection of PAH in water, which is in agreement with the idea of balanced development, may be useful for improving scientific research and implementation of new technologies.

### Evaluation of Detection Limits of the Luminescence and Electrochemiluminescence Methods

The detection limits of the luminescence and electroluminescence methods were determined on the basis of the signal to noise ratio:

$$ DL=\frac{Is}{I_N} $$

where I_S_ is the mean intensity of the signal of the sample (emission intensity in arbitrary units) and I_N_ is the mean intensity of the signal of the background.

The noise was assumed to be the emission intensity for the sample without PAHs. The usually assumed detection limit is the concentration of the component to be determined at the level at which $\frac{I_S}{I_N}=3$. For phenanthrene, pyrene, and naphthalene, the detection limit of electroluminescence method was 3.0, 0.2, and 10.0 μg/dm^3^, respectively. The corresponding values of the fluorescence method were similar: 2.0, 0.7, and 18.0 μg/dm^3^.

### Metagenomic Analysis of S4 Bacterial Consortium

**Fig. 5:**
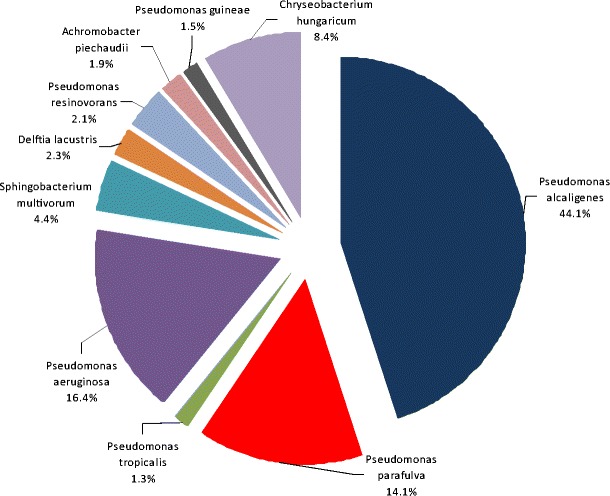
Top species classification results of bacterial consortium S4

In order to identify the S4 bacterial consortium, ametagenomic analysis of the gene encoding 16S rRNA was conducted on the basis of V4 hypervariable region of the 16S rRNA gene. This allowed to identify 198 species of bacteria, which create the S4consortium. Preparation of the reference database of the reference sequences included filtering the sequences with a length shorter than 1250 bp, sequences containing more than 50 degraded bases and partially classified sequences, which could not be attributed to genus or species level. After the sequencing statistics, a total of 99.347 reads were obtained, 95.889 of which remained after passing quality filtering (which accounted for 96.5 % reads passing quality filtering). Bacteria dominated in the consortium (99.14 %), which consisted of *Gammaproteobacteria* (78.82 %), *Flavobacteria* (9.25 %), *Betaproteobacteria* (7.68 %), *Sphingobacteria* (3.76 %), *Alphaproteobacteria* (0.42 %), *Clostridia* (0.04 %), and *Bacilli* (0.03 %). The total number of identified species-level taxonomic categories was at 198. Figure 5 shows the top 10 of 198 classifications. The “Other” category in this pie chart is the sum of all remaining classifications (188) with less than 3.5 % abundance (Table 1).

**Table 1:** The “Other” category in this pie chart is the sum of all classifications (198) with less than 3.5 % abundance

Taxonomic level: species	Relative abundance (%)	Taxonomic level: species	Relative abundance (%)	Taxonomic level: species	Relative abundance (%)
*Achromobacter arsenitoxydans*	0,034414	*Gallionella ferruginea*	0,001042	*Pseudomonas lundensis*	0,219003
*Achromobacter denitrificans*	0,001042	*Gemella sanguinis*	0,001042	*Pseudomonas mendocina*	0,003128
*Achromobacter insolitus*	0,269061	*Haemophilus parainfluenzae*	0,002085	*Pseudomonas metavorans*	0,009385
*Achromobacter xylosoxidans*	0,019814	*Halorhodospira halochloris*	0,003128	*Pseudomonas moraviensis*	0,003128
*Acidovorax anthurii*	0,001042	*Klebsiella pneumoniae*	0,004171	*Pseudomonas oleovorans*	0,002085
*Acidovorax facilis*	0,001042	*Lachnospira pectinoschiza*	0,001042	*Pseudomonas oryzihabitans*	0,005214
*Acidovorax wohlfahrtii*	0,013557	*Lactobacillus letivazi*	0,001042	*Pseudomonas otitidis*	0,678910
*Acinetobacter antiviralis*	0,003128	*Lactobacillus plantarum*	0,001042	*Pseudomonas panacis*	0,004171
*Acinetobacter ursingii*	0,001042	*Lactobacillus taiwanensis*	0,001042	*Pseudomonas panipatensis*	0,011471
*Agrobacterium larrymoorei*	0,002085	*Lautropia mirabilis*	0,006257	*Pseudomonas plecoglossicida*	0,022943
*Agrobacterium tumefaciens*	0,071958	*Leptothrix cholodnii*	0,001042	*Pseudomonas poae*	0,075086
*Anaerococcus lactolyticus*	0,002085	*Leptothrix discophora*	0,006257	*Pseudomonas proteolytica*	0,032329
*Anaerococcus tetradius*	0,001042	*Leptotrichia buccalis*	0,001042	*Pseudomonas pseudoalcaligenes*	0,016686
*Aquitalea denitrificans*	0,001042	*Limnobacter litoralis*	0,001042	*Pseudomonas putida*	0,028157
*Azorhizophilus paspali*	0,008343	*Marinobacter santoriniensis*	0,001042	*Pseudomonas rhodesiae*	0,330590
*Bacteroides ovatus*	0,001042	*Marinobacter szutsaonensis*	0,001042	*Pseudomonas straminea*	0,001042
*Bradyrhizobium japonicum*	0,003128	*Marinomonas protea*	0,002085	*Pseudomonas stutzeri*	0,240903
*Bradyrhizobium pachyrhizi*	0,003128	*Methylobacterium persicinum*	0,001042	*Pseudomonas syncyanea*	0,029200
*Brevundimonas bullata*	0,003128	*Microbulbifer cystodytense*	0,003128	*Pseudomonas synxantha*	0,003128
*Brevundimonas diminuta*	0,001042	*Microlunatus ginsengisoli*	0,001042	*Pseudomonas teessidea*	0,022943
*Burkholderia phenoliruptrix*	0,001042	*Mitsuaria chitosanitabida*	0,001042	*Pseudomonas thermotolerans*	0,002085
*Butyrivibrio proteoclasticus*	0,002085	*Nitrincola lacisaponensis*	0,006257	*Pseudomonas tolaasii*	0,001042
*Candidatus Blochmannia rufipes*	0,017728	*Nitrobacter vulgaris*	0,001042	*Pseudomonas tremae*	0,911470
*Candidatus Phlomobacter fragariae*	0,001042	*Nitrobacter winogradskyi*	0,001042	*Pseudomonas tropicalis*	1,324448
*Cellulomonas denverensis*	0,001042	*Ochrobactrum anthropi*	0,007300	*Pseudomonas veronii*	0,011471
*Chryseobacterium caeni*	0,002085	*Ochrobactrum intermedium*	0,011471	*Pseudomonas xanthomarina*	0,010428
*Chryseobacterium taeanense*	0,214831	*Ochrobactrum pecoris*	0,003128	*Pseudoxanthomonas sacheonensis*	0,004171
*Chryseobacterium ureilyticum*	0,001042	*Ochrobactrum thiophenivorans*	0,234646	*Rhodoferax antarcticus*	0,003128
*Citrobacter freundii*	0,003128	*Oxalobacter vibrioformis*	0,001042	*Roseateles depolymerans*	0,002085
*Citrobacter werkmanii*	0,002085	*Pedomicrobium manganicum*	0,003128	*Salmonella enterica*	0,001042
*Clostridium frigoris*	0,001042	*Pelomonas puraquae*	0,001042	*Serratia entomophila*	0,001042
*Clostridium perfringens*	0,001042	*Peptoniphilus asaccharolyticus*	0,001042	*Serratia symbiotica*	0,001042
*Comamonas kerstersii*	0,001042	*Peptoniphilus coxii*	0,001042	*Shinella yambaruensis*	0,001042
*Corynebacterium amycolatum*	0,004171	*Peptoniphilus lacrimalis*	0,001042	*Slackia exigua*	0,001042
*Corynebacterium aurimucosum*	0,001042	*Peptoniphilus methioninivorax*	0,001042	*Sphingobacterium siyangense*	0,281575
*Corynebacterium coyleae*	0,002085	*Photobacterium aquimaris*	0,001042	*Sphingobacterium thalpophilum*	0,004171
*Corynebacterium jeikeium*	0,001042	*Pigmentiphaga daeguensis*	0,002085	*Staphylococcus aureus*	0,003128
*Corynebacterium matruchotii*	0,001042	*Polaromonas ginsengisoli*	0,001042	*Staphylococcus caprae*	0,002085
*Corynebacterium riegelii*	0,002085	*Polynucleobacter rarus*	0,001042	*Stenotrophomonas acidaminiphila*	0,036500
*Corynebacterium xerosis*	0,001042	*Porphyromonas asaccharolytica*	0,001042	*Stenotrophomonas chelatiphaga*	0,050057
*Cupriavidus basilensis*	0,037543	*Porphyromonas bennonis*	0,005214	*Stenotrophomonas maltophilia*	0,344147
*Cupriavidus campinensis*	0,009385	*Porphyromonas canis*	0,001042	*Stenotrophomonas pavanii*	0,018771
*Cupriavidus pauculus*	0,034414	*Porphyromonas somerae*	0,003128	*Stenotrophomonas retroflexus*	0,208574
*Curvibacter gracilis*	0,031286	*Porphyromonas uenonis*	0,006257	*Stenotrophomonas terrae*	0,005214
*Delftia acidovorans*	0,318076	*Prevotella melaninogenica*	0,003128	*Streptococcus agalactiae*	0,001042
*Delftia tsuruhatensis*	0,474507	*Prevotella timonensis*	0,001042	*Streptococcus fryi*	0,001042
*Desulfovibrio ferrophilus*	0,001042	*Prevotella veroralis*	0,002085	*Streptococcus milleri*	0,001042
*Dialister invisus*	0,001042	*Propionibacterium acnes*	0,004171	*Streptococcus oralis*	0,002085
*Dokdonella fugitiva*	0,144959	*Pseudomonas alcaliphila*	0,030243	*Streptococcus pseudopneumoniae*	0,002085
*Dysgonomonas wimpennyi*	0,002085	*Pseudomonas argentinensis*	0,001042	*Streptococcus tigurinus*	0,002085
*Ectothiorhodospira haloalkaliphila*	0,008343	*Pseudomonas azotoformans*	0,443220	*Streptococcus vestibularis*	0,003128
*Enterobacter aerogenes*	0,002085	*Pseudomonas benzenivorans*	0,300347	*Sutterella sanguinus*	0,002085
*Enterobacter amnigenus*	0,619466	*Pseudomonas brenneri*	0,058400	*Thermomonas brevis*	0,001042
*Enterobacter cancerogenus*	0,001042	*Pseudomonas chloritidismutans*	0,004171	*Thioalkalimicrobium sibiricum*	0,002085
*Enterobacter cloacae*	0,009385	*Pseudomonas coronafaciens*	0,002085	*Thiomonas thermosulfata*	0,001042
*Enterobacter gergoviae*	0,001042	*Pseudomonas cremoricolorata*	0,002085	*Tolumonas auensis*	0,009385
*Enterobacter hormaechei*	0,045886	*Pseudomonas fluorescens*	0,009385	*Variovorax boronicumulans*	0,007300
*Enterobacter ludwigii*	0,002085	*Pseudomonas fragi*	0,045886	*Variovorax ginsengisoli*	0,001042
*Erwinia tasmaniensis*	0,001042	*Pseudomonas fulva*	0,006257	*Vogesella perlucida*	0,004171
*Filifactor villosus*	0,009385	*Pseudomonas gessardii*	0,001042	*Yersinia frederiksenii*	0,017728
*Fusobacterium gonidiaformans*	0,003128	*Pseudomonas gingeri*	0,001042	*Yersinia massiliensis*	0,011471
*Fusobacterium nucleatum*	0,001042	*Pseudomonas jessenii*	0,001042	*Yersinia pseudotuberculosis*	0,003128
*Fusobacterium periodonticum*	0,001042				

## Submission

The carried out PAH biodegradation kinetics measurements in the aqueous environment with the use of the S4 bacterial consortium indicate that the efficiency of the biodegradation process was high and that aromatic hydrocarbons may be successfully removed even at high concentrations. It is commonly believed that only the dissolved fraction of PAH is bioavailable for the microorganisms. As such, the evaluation of the biotechnological potential with respect to bioremediation may be carried out directly on the basis of the changes in the concentrations of water soluble fractions. The employed analysis combining ECL and FL confirms this possibility. In both cases, it was unequivocally established that naphthalene was the most biodegradable PAH, whereas pyrene was most resistant to biodegradation. The half-life times of naphthalene, phenanthrene, and pyrenewere at 35, 75, and 130 h, accordingly. It was concluded that these differences are directly related to various solubility limits of the studied compounds. Upon adequate modifications of the bioreactor, the high voltage ECL as a method for determination of changes in the concentrations of water soluble PAH fractions may be used for on-line monitoring of changes in PAH concentrations without the need of sample collection.
